# Study of Size Effect on Microstructure and Mechanical Properties of AlSi10Mg Samples Made by Selective Laser Melting

**DOI:** 10.3390/ma11122463

**Published:** 2018-12-04

**Authors:** Zhichao Dong, Xiaoyu Zhang, Wenhua Shi, Hao Zhou, Hongshuai Lei, Jun Liang

**Affiliations:** 1 Institute of Advanced Structure Technology, Beijing Institute of Technology, Beijing 100081, China; zc339580@126.com; 2 Beijing Key Laboratory of Intelligent Space Robotic Systems Technology and Applications, Beijing Institute of Spacecraft System Engineering, Beijing 100094, China; xiaoyuzh.001@hotmail.com (X.Z.); nafer@126.com (W.S.); 3 State Key Laboratory of Explosion Science and Technology, Beijing Institute of Technology, Beijing 100081, China

**Keywords:** size effect, X-ray computed tomography, microstructure, mechanical properties, selective laser melting

## Abstract

The macroscopic mechanical performance of additive manufactured structures is essential for the design and application of multiscale microlattice structure. Performance is affected by microstructure and geometrical imperfection, which are strongly influenced by the size of the struts in selective laser melting (SLM) lattice structures. In this paper, the effect of size on microstructure, geometrical imperfection, and mechanical properties was systemically studied by conducting experimental tests. A series of AlSi10Mg rod-shaped samples with various diameters were fabricated using SLM. The uniaxial tensile test results show that with the decrease in build diameter, strength and Young’s modulus of strut decreased by 30% more than the stable state. The main reasons for this degradation were investigated through microscopic observation and micro X-ray computed tomography (μ-CT). In contrast with large-sized strut, the inherent porosity (1.87%) and section geometrical deviation (3%) of ponysize strut is greater because of the effect of thermal transform and hydrogen evolution, and the grain size is 0.5 μm. The discrepancy in microstructure, geometrical imperfection, and mechanical properties induced by size effect should be considered for the design and evaluation of SLM-fabricated complex structures.

## 1. Introduction

Additive manufacturing (AM), as a developed rapid manufacturing technique, has been widely used to produce complex-shaped or multiscale ultralight metallic structures such as microlattice structures [1,2,3,4,5]. Among these AM technologies, selective laser melting (SLM) is popular because of its high level of degree of freedom in manufacturing; SLM enables the layer-by-layer production of metal parts based on computer-aided design (CAD) data [6,7,8]. In terms of cellular structures, the lattice structures consist of a connected framework of struts. The strut is regarded as a typical unit of cells in lattice structures, and the structure deforms by the stretching of the struts. Therefore, the safety design and evaluation of lattice structures significantly depend on the microstructure heterogeneities, geometric accuracy, and mechanical properties of struts, which are usually caused by AM factors, such as build size. The build size of lattice struts varies from micron to millimeter [6,9,10]. In fact, the microstructure and mechanical properties of built struts clearly depend on the manufacturing process parameters [11] including the build orientation, heating treatments, and scanning speed. The precise geometry and performance of struts are important for the safety design and evaluation of lattice structures [7,8,11].

Recently, many studies have been conducted to investigate the effects of build direction and processing parameters on the microstructure and mechanical properties of build microstruts [12,13,14,15]. Hadadzadeh et al. [11] evaluated the effect of build direction on the high strain rate dynamic loading behavior of AlSi10Mg rod-shaped samples of 12 mm diameter. The experimental results showed that significant differences in overall modulus and strength exist owing to the distinct cell structure, grain size, and dislocation density. Delroisse et al. [10] compared microstructure heterogeneity and hardness between inclined and vertical struts of AlSi10Mg lattices fabricated using SLM. Hitzler et al. [12] reported the directional dependencies of SLM-processed AlSi10Mg parts. The experimental results showed that tensile strength can be improved by 27%, and the elastic modulus (EM) increased by 17% in different building directions. They reported the microstructure and mechanical behavior with the strut orientation relative to the building direction. Moreover, Thijs et al. [13] reported that the microstructure can be controlled, and it depends on the SLM process parameters. Li et al. [14] proved the effect of applied process parameters on the densification and mechanical performance of parts. Wei et al. [15] systematically evaluated the effects of process parameters (laser power, scanning speed, and hatch spacing) on the densification level and mechanical properties of bulk AlSi10Mg alloy samples produced using SLM. However, the influence of the size of the struts has rarely been studied. Thus, the effects of microstructure and geometrical imperfection remain unclear.

Some studies show that geometrical imperfection is an important factor affecting the mechanical properties of SLM-fabricated struts, including the built deviation and interior porosity. Delroisse et al. [10] reported the effects of the orientation and spatial location of struts on the microstructure and porosity. Takanao et al. [16] studied the effects of the geometrical imperfection of struts on the compressive response of lattice blocks using experimental tests and the finite element method (FEM). A stochastic homogenization analysis model was proposed by considering the uncertainty in mechanical property induced by geometrical imperfection. Significant differences in the microstructure and porosity exist between different zones. Lhuissier et al. [17] established a cellular automaton model to predict the morphological evolution of an as-built strut and evaluated the effect of defects on the elastic mechanical properties of struts. In fact, the macroscopic mechanical performances of built structures of various sizes might be different owing to the particular nature of SLM. Suard et al. [18] found significant size differences between the designed and manufactured struts by measuring the equivalent size of struts. The results show that size discrepancies would degrade the stiffness of struts. Nava et al. [19] evaluated the effects of density and feature size on the mechanical properties of isostructural metallic foams. Yan et al. [20] evaluated the effect of unit cell size on the manufacturability and mechanical performance of lattice structures. Tancogne-Dejean et al. [21] evaluated the tensile performance of SLM-fabricated cylindrical samples with diverse build sizes, and the results show that the yield strength (YS) decreased by 50% when the sample size was decreased from 8 mm to 0.8 mm.

High-fidelity X-ray microcomputed tomography (μ-CT) technology has been gradually used as a nondestructive testing method for defect detection and mesostructure reconstruction of complex metal or composite structures [8,16,18,20,22], which can accurately obtain the morphology and microdefect of struts. Leary et al. [23] and Takano et al. [16] investigated the geometrical imperfection or irregularity of lattice struts using μ-CT. Alsalla et al. [24] evaluated the effect of manufactured defects on the tensile properties and fracture toughness of the cellular structure. Kim et al. [25] developed a feedback mechanism that improved the initial design by using the μ-CT technique to quantify the surface roughness and pore size.

In summary, a review of previous studies shows that the process parameters of SLM significantly affect the microstructure’s heterogeneity and macroscopic performance of fabricated structures, especially for microlattice structures. Nevertheless, these studies mainly focused on the build direction, scanning speed, and parent materials. To the best of our knowledge, the influence of size effect on the microstructure, geometrical imperfection, and mechanical properties of strut has rarely been studied.

The aim of this study was to investigate the influence of size effect on the microstructure, geometrical imperfection, and mechanical properties of SLM-fabricated AlSi10Mg lattice struts, essential for further design and application of multiscale metallic structures. A series of rod-shaped samples with various build sizes were prepared using SLM with the same process parameters. The microstructure, fracture morphologies, geometrical features, and mechanical properties were systematically characterized using scanning electron microscope (SEM), X-ray microcomputed tomography, and uniaxial tensile tests.

## 2. Materials and Methods

### 2.1. Material and Manufacturing

**Figure 1:**
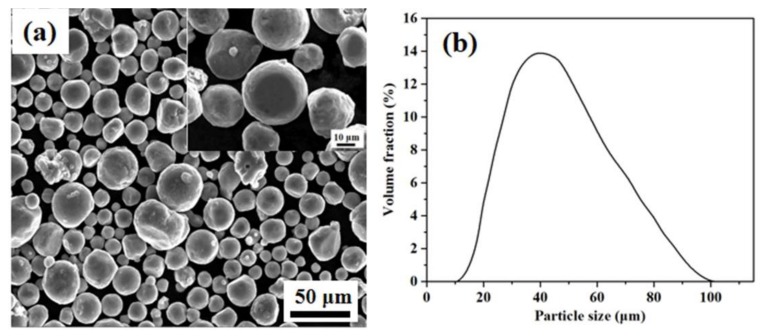
Scanning electron microscope (SEM) image of starting powders: (**a**) low magnification and high magnification (inset); (**b**) AlSi10Mg particle size distributions.

In this study, AlSi10Mg alloy powder was used as the parent material, which has been extensively used in aerospace and automate sectors because of its good castability and corrosion resistance [26]. The average diameter of particles is 35 µm, and the nominal chemical composition is shown in Table 1. Figure 1a shows the microtopography of powders observed using a scanning electron microscope (SEM) at low and high magnifications. The particle size distribution of D10: 22.13 μm, D50: 37.12 μm, D90: 62.45 μm is shown in Figure 1b. This was measured using a Hydro 2000MU (A) laser particle size instrument.

**Figure 2:**
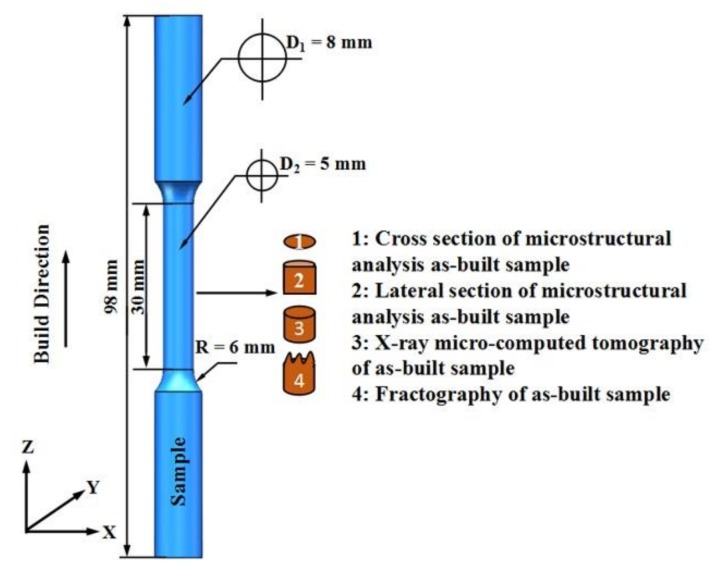
Schematic of tensile samples (diameter of 5 mm) used for microstructure, fractography analysis, and μ-CT scanning.

Rod-shaped samples with various sizes (1, 2, 3, 4 and 5 mm) were fabricated by SLM using a Concept X-line 1000R machine (Hofmann, Lichtenfels, Germany) with an available building space of 250 × 250 × 325 mm^3^ volume. The geometrical size and built direction of samples obtained using the same process parameters are shown in Figure 2. Due to the constraints of the SLM process, it is difficult to fabricate horizontal strut of cells in lattice structure. Therefore, the long axis of samples was orientated parallel to the building direction. The SLM system has an Yb-fiber laser with 400 W power and a 100 μm laser spot size. All the processing was performed in an argon atmosphere with an oxygen content of <0.1%. The laser power was 370 W, and the hatch spacing was 190 μm. The scanning speed and layer thickness were set at 1500 mm/s and 30 μm, respectively, as determined by the optimization of process parameters. A laser scanning strategy was used; the laser beam was rotated at 67° between the continuous layers. In the entire SLM process, the temperature of the substrate plate was kept constant at 35 °C.

### 2.2. Experimental Tests

To evaluate the manufacturing quality of as-built samples, the μ-CT technique was used to accurately obtain the structural morphology and internal porosity using a GE Phoenix v|tome|x 240 laboratory system (225 kv, GE, Phoenix, AZ, USA). The source voltage and tube current of the system were set as 100 kV and 210 µA. The samples were fixed on a rotatable platform, and the rotating speed was 0.36°/s for each rotation step. Thus, 1000 data profiles were acquired and imported into the Mimics 20.0 commercial software to reconstruct the actual three-dimensional (3D) topographies. The resolution of μ-CT was 2048 × 2048 pixels and the isotropic voxel size was (4 µm)^3^. The geometric imperfection and internal void defects of pretest samples were extracted and analyzed.

The microstructure of as-built samples was studied using an Axio Scope A1 optical microscope (OM, Zeiss, Jena, Germany) and SU8010 SEM (SEM, HITACHI, Tokyo, Japan). The main aim was to observe the variation in microstructure, including the grain size and overlapped melt pools. Two types of blocks were cut from the samples along the vertical and horizontal directions. These blocks were ground, polished, and etched using a reagent (2 mL HF, 3 mL HCl, 5 mL HNO_3_, and 190 mL H_2_O) for 25 s. Moreover, the fracture surfaces of samples after the tensile test were observed using SEM.

**Figure 3:**
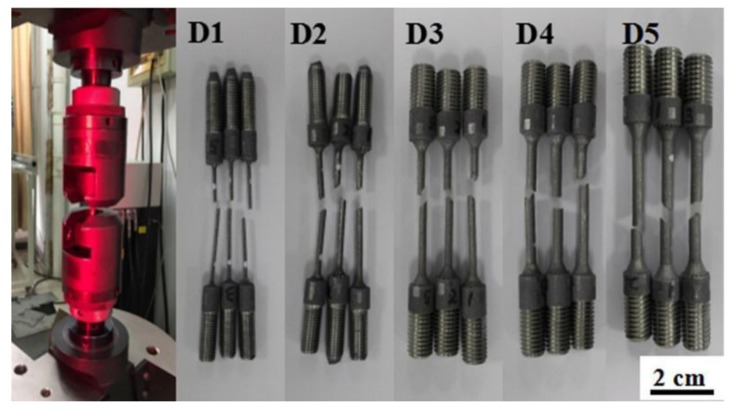
Uniaxial tensile test setup and selective laser melting (SLM)-fabricated AlSi10Mg samples.

Uniaxial tensile tests were conducted at room temperature using an Instron 5985 universal machine according to the methods used from [27], and the crosshead speed was set at 1.0 mm/min. The axial displacement was measured using an infrared video extensometer (Instron AVE 2.0, INSTRON, Boston, MA, USA), and its precision was 0.5 per thousand. The specific experimental setup and SLM-built tensile samples are shown in Figure 3. These samples were named as D1, D2, D3, D4 and D5 corresponding to various build sizes.

## 3. Results and Discussion

### 3.1. Microstructural Analysis

**Figure 4:**
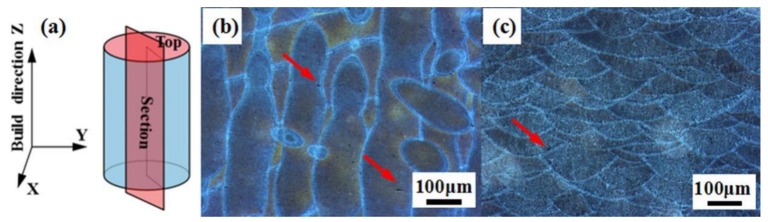
Optical micrographs of D4 sample: (**a**) building direction and sectioning for microscopy; (**b**) top view; (**c**) section view. (Red arrow is used to mark the pores).

The optical micrographs of the sample (D4) in the top and section views are shown in Figure 4. The geometry, arrangement, and boundaries of melt pools can be clearly observed, as indicated by references [28,29]. According to the microstructure, a scanning strategy (67° rotation of laser beam between continuous layers) was used to observe the overlapped melt pools and the pores with various sizes and shapes. The presence of small pores (as shown by red arrows in Figure 4b,c) can be attributed to hydrogen evolution induced porosity, the remnant of shield gas in the melt pool during SLM, and the pores inside the initial powders transferred to the as-built sample [29,30]. In fact, the optimized process parameters produce a strong bonding between adjacent scanning tracks and layers in the as-built sample. In addition, the lack of sufficient energy for complete melting or spatter ejection may form pores during SLM [29]. The pores can deteriorate the mechanical performance of as-built structures.

**Figure 5:**
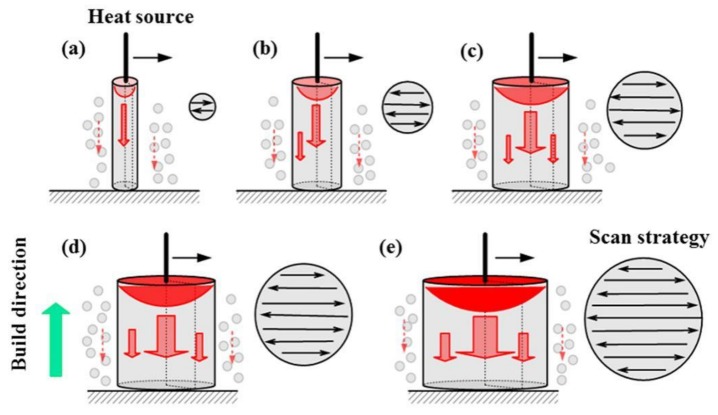
Schematic representation of thermal transfer and the corresponding scan strategy during SLM for different build sizes: (**a**) 1 mm; (**b**) 2 mm; (**c**) 3 mm; (**d**) 4 mm and (**e**) 5 mm. Red arrows show the thermal flux density and direction.

The thermal transfer history during SLM strongly affects the microstructure and mechanical properties of as-built samples. Figure 5 shows the schematic of the thermal transfer effect and corresponding scan strategies for various build sizes. The black lines denote the laser beam, and the red regions represent the consequent melt pool. The red arrows denote the thermal transfer (density and direction) process. The as-built samples are surrounded by sintered powder. It was observed that the middle part of the thick strut fully melted because of the heat accumulation effects during multiple scanning processes. This is the main reason for excellent mechanical properties.

**Figure 6:**
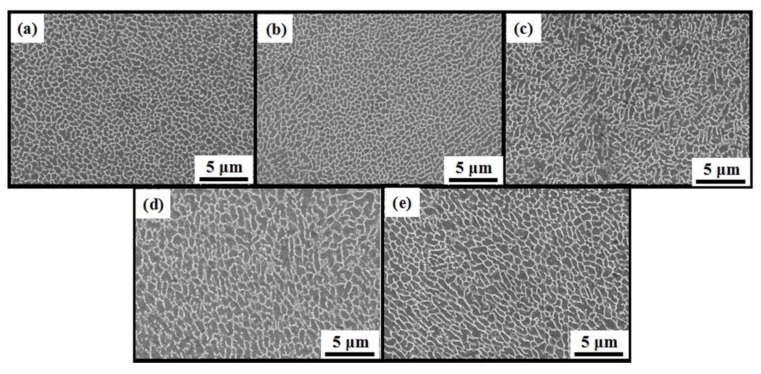
SEM images of as-built AlSi10Mg samples for different build sizes: (**a**) 1 mm; (**b**) 2 mm; (**c**) 3 mm; (**d**) 4 mm and (**e**) 5 mm. They show the cellular microstructure of the center of the melt pool.

The SEM images of as-built strut samples with various build sizes are shown in Figure 6. The microstructure of the melt pool center was characterized with a fine cellular–dendritic microstructure in which a rich eutectic silicon network is distributed in the Al-matrix [31]. As shown in Figure 6, the morphologies of the cells of the melt pool center are different as the build size changes. For the thicker rods, a coarser and elongated microstructure was observed in contrast to a fine equiaxed grain in the small rods. This indicates that columnar-to-equiaxed transition occurred in the microstructure with the change in build size from 5 mm to 1 mm. Figure 6a,b shows that the melt pool center consists of equiaxed grain with cell sizes in the range from 0.5 μm to 1 μm. It can be considered that the relatively small reaction area can weaken the heat accumulation (as shown in Figure 5a,b). For a given area, the extremely low number of scanning tracks indicates a lack of laser energy inputs, which may increase the cooling rate and refine the grain [32]. D4 and D5 samples showed a coarser and elongated structure in contrast to that observed for D1 and D2 samples, and the average cell size increased from 0.75 μm to 1.75 μm. The detailed values of microstructure cell sizes are shown in Table 2.

The coarser grain can be related to a different thermal history across the melt pool. For the given process parameters, the laser energy absorbed per unit area is invariable. When a large reaction area is sintered for SLM, regions of overmelting and pores arise between the neighboring tracks owing to the increase in heat accumulation. In addition, the regions around the melt pool boundaries or at overlaps between the melt pools may remain at higher temperatures for longer times, resulting in a slower solidification rate and consequently a coarser microstructure [33,34].

### 3.2. Geometrical Imperfection Analysis

**Figure 7:**
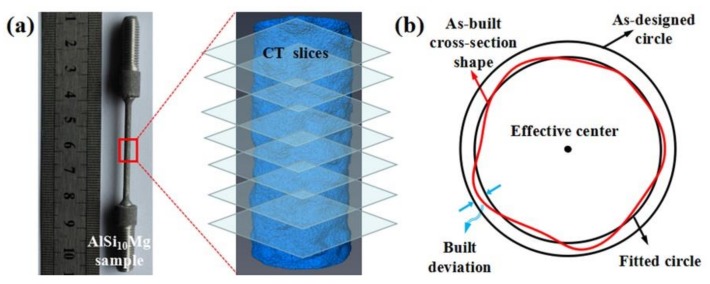
Schematic of CT image extraction process: (**a**) sample and CT slices; (**b**) built deviation and as-designed values.

Geometrical imperfection is an important factor affecting the mechanical properties of SLM-fabricated struts, such as the built deviation and interior porosity [16,35]. Liu et al. [35] reported the geometric deviation of an as-fabricated sample reconstructed from CT images. For a representative strut, the CT image extraction process used to acquire the morphological defects is shown in Figure 7. The geometry of samples was acquired and discretized with CT slices. A series of parallel slices were created to intersect the sample at equidistance along the strut axis. On each slice, one of which is shown in Figure 7b, the shape boundary of a cross-section of the sample can be described using a series of circles (as-built, as-designed, and fitted circles). The discrepancy between the build size of a fitted circle and an as-designed circle can be determined, thus evaluating the built deviation from that of the fitted circle.

**Figure 8:**
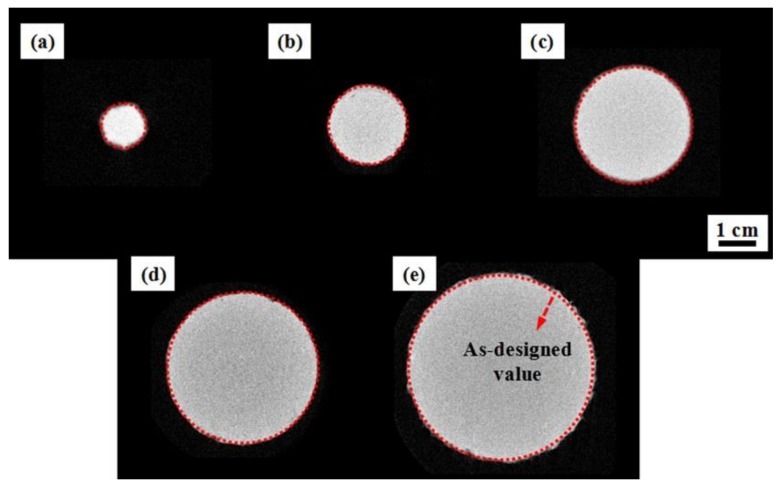
Two-dimensional (2D) segmented CT slices of cross-sections of AlSi10Mg samples for different build sizes: (**a**) 1 mm; (**b**) 2 mm; (**c**) 3 mm; (**d**) 4 mm and (**e**) 5 mm. (Red dotted circles indicate the design values).

Figure 8 shows the obtained two-dimensional (2D) CT scans of AlSi_10_Mg samples with various build sizes before the tensile tests. In this study, a relatively conservative method was used to describe the as-built cross-section shape, as mentioned above. By comparison, it can be found that the section areas of thin rods (D1, D2, and D3) are slightly lower than the as-designed values (marked by red dotted circles). Nevertheless, the effective section sizes of D4 and D5 samples are almost identical to the designed value. To obtain the quantitative deviation, 500 CT slice images were used to rebuild the 3D real geometrical configurations using Mimics software. The selected 3D reconstructed strut is appropriate and representative in capturing deviation distribution.

**Figure 9:**
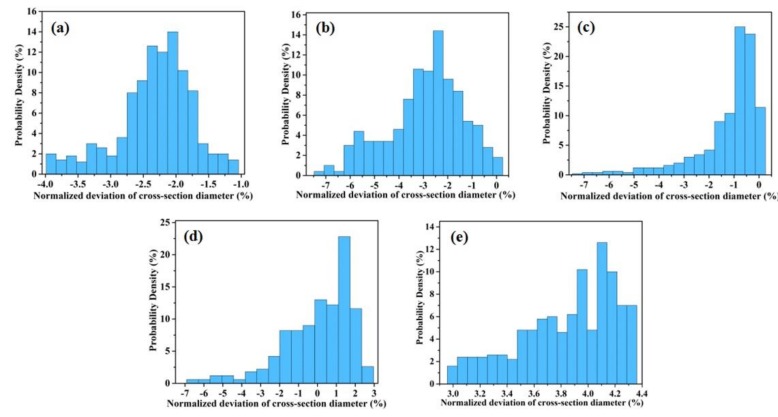
Probability density distributions of SLM normalized deviation for struts with different build sizes: (**a**) 1 mm; (**b**) 2 mm; (**c**) 3 mm; (**d**) 4 mm and (**e**) 5 mm.

Figure 9 shows the probability density distributions of built deviation normalized by the nominal values of as-designed size for the rod samples. The size deviation of D1 is mainly concentrated in the range from −4.0% to −1%, and the average deviation is about −2.8%. The maximum probability density is about 14% (−2.0%). For the D5 sample, the deviation range is from 3.0% to 4.3%. The average deviation and probability density are ~3.9% and 13% (4.1%), respectively. With the increasing of build diameter, the surface roughness of the samples is gradually reduced. The size variation will affect the precision of overall strength and modulus determined by tests. Moreover, the results show that the surface of D1 and D2 is fairly rough in contrast with the other types. In previous study [36], the experimental results showed that the compressive stiffness and strength of porous structures decreased by ~80% when the strut size decreased by ~50%. This indicates that the strength of the lattice structure depends on the geometrical size of struts.

**Figure 10:**
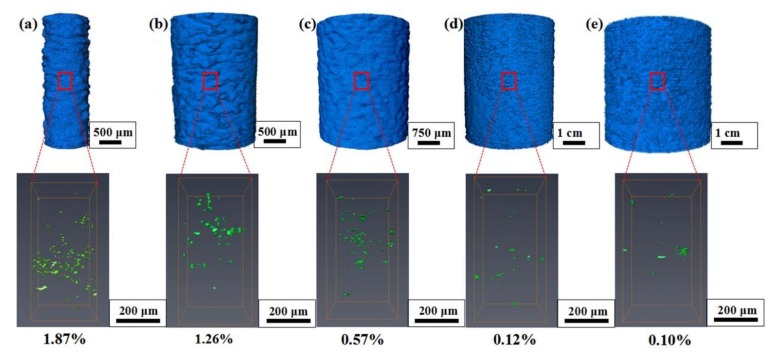
Morphology and porosity of vertical struts for different build sizes: 3D reconstruction structure of struts (blue) and pores (green). (**a**) 1 mm; (**b**) 2 mm; (**c**) 3 mm; (**d**) 4 mm and (**e**) 5 mm.

The internal void defect of samples was also estimated based on the 3D reconstructed model, as shown in Figure 10. According to the statistical results in the respective volumes (90 × 80 × 180 voxel^3^), we found that the porosity of the sample increased significantly with the decrease in build size. The specific values of porosity are listed in Table 3. This phenomenon is consistent with the previous analysis of thermal transform. The main cause is related to the thermal transfer history in SLM due to a high input of laser energy. The fewer scan tracks indicate the lack of laser energy inputs, which may lead to insufficient melting and pore formation. With the increase in build size, the increasing scanning tracks and sintered area can provide high energy inputs (see Figure 5). A similar level of porosity (from 0.1% to 4%) was reported by Qiu et al. [37] for AlSi_10_Mg alloy struts estimated by μ-CT. The other cause for the generation of pores in AlSi_10_Mg alloy samples is the hydrogen evolution in SLM techniques [10,38,39]. The generation of an inherent void will affect the mechanical performance of SLM-fabricated structures.

### 3.3. Mechanical Properties of SLM-Built Samples

**Figure 11:**
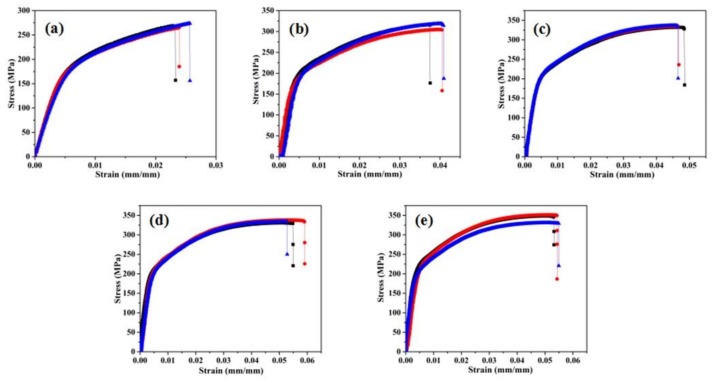
Stress–strain curves of as-built AlSi10Mg uniaxial tensile samples for different build sizes: (**a**) 1 mm; (**b**) 2 mm; (**c**) 3 mm; (**d**) 4 mm and (**e**) 5 mm.

**Figure 12:**
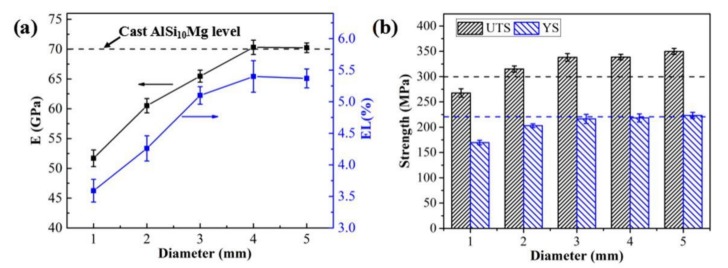
Effect of build size on the mechanical performance of AlSi10Mg samples: (**a**) EM and EL; (**b**) UTS and YS.

The typical stress–strain curves of the SLM-fabricated AlSi_10_Mg alloy samples containing three effective curves for each category obtained from uniaxial tensile tests are shown in Figure 11. The parent material exhibited an excellent elastic-plastic response behavior, similar to that obtained using a traditional casting process [28]. The mechanical parameters can be determined from these curves, including the elastic modulus (EM), ultimate tensile strength (UTS), YS, and elongation (EL). The specific values are shown in Figure 12 and Table 4. Significant differences were observed in the mechanical properties of these samples owing to the variation in build size. The macroscopic mechanical parameters maintained stable values (~70 GPa, 5.5%, 345 MPa, and 220 MPa) when the build size exceeded 4 mm. The UTS and YS of SLM-built samples achieved the level of casting (300 MPa, 220 MPa) or slightly higher [28]. However, with the decrease in built size from 5 mm to 1 mm, the EM and EL of samples decreased by >30%, and the strength (UTS and YS) decreased by ~24%. This phenomenon is different from the experimental results obtained for traditional solid materials, indicating that the design of a multiscale lattice structure should consider the discrepancy in material property scattering to the variation in print size. In previous studies, the main reasons for the degradation of materials were the appearance of inherent defects (unmelted particles and pores), as confirmed by microstructure characterization [18,40,41]. This phenomenon can also be explained by the conclusions of the previous Section 3.1 and Section 3.2.

### 3.4. Fractography Analysis

**Figure 13:**
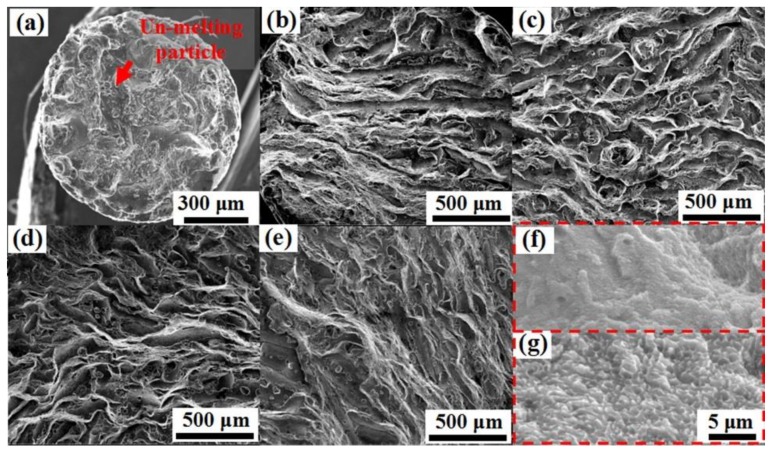
SEM images of fracture surface morphologies of AlSi10Mg samples (D1, D2, D3, D4, and D5) for different build sizes: (**a**) 1 mm; (**b**) 2 mm; (**c**) 3 mm; (**d**) 4 mm; (**e**) 5 mm. (**f**) and (**g**) show the local high-magnification images of (a) and (d), respectively.

To further elucidate the mechanism of degradation, the fracture surface morphologies of SLM-fabricated AlSi_10_Mg alloy samples are shown in Figure 13. With the increase in build size, the characteristics of fracture gradually transform from brittle fracture into brittle-ductile fracture. The unmelted particles, cracks, and dimples, typical brittle-ductile types of fracture features, can be observed on the fracture surfaces of samples D1–D5 (Figure 13a–e). This indicates that the build size significantly affects the fracture mechanism of the samples. The fracture occurs throughout the scan tracks and leads to complete or partial separation from the scan track boundaries [42]. Furthermore, the high-magnification images of samples D1 and D4 are shown in Figure 13f,g, respectively. Here, brittle fracture features (flat planes or cleavage surfaces) are observed for sample D1, whereas brittle-ductile fracture features (elongated dimples of almost equal (~1 µm) size) are observed for sample D4. In the fracture surface of sample D4, dimples, a sign of ductile fracture, are more visible than that of sample D1, consistent with the higher EL result obtained for sample D4 (see Figure 11 and Figure 12). As a result, the crack initiation and propagation started with the nucleation of pores, followed by their growth and coalescence. Thus, it can be concluded that porosity can lead to the deterioration of ductility of samples.

## 4. Conclusions

In this study, we investigated the influence of size effect on the microstructure, geometrical imperfection, and macroscopic mechanical properties of SLM-prepared AlSi_10_Mg lattice strut. The following conclusions are drawn:

- The microstructure of SLM-processed AlSi10Mg lattice strut depends strongly on the build size of struts. For subsize struts, the equiaxed grain can be observed, and the grain average size is 0.75 μm. This contrasts to the coarser and more elongated (approximately 1.75 μm) microstructure that was observed in the large-sized samples.
- The size effect has a significant influence on the geometric imperfection for SLM-processed AlSi10Mg strut. With the decrease of build size, the inherent void defect and geometrical deviation will apparently increase owing to the effects of thermal transform and hydrogen evolution during SLM. The porosity level of the sample decreased from 1.87% to 0.1% with the variation of build size from 1 mm to 5 mm.
- The experimental results show that overall strength and modulus significantly decrease by ~30% with the decrease in build size. In the fracture surface of large-sized strut, tiny dimples, a sign of ductile fracture, are more visible than that of ponysize strut.
- These findings show that the discrepancies in microstructure, geometrical imperfection, and mechanical performances due to size effect should be considered for the design and application of multiscale structures.

## Figures and Tables

**Table 1:** Chemical composition of AlSi10Mg alloy powder.

**Element**	Al	Si	Mg	Fe	Mn	Ni	Ti	Cu
**Composition (wt %)**	Balance	10.1	0.45	0.4	0.5	0.05	0.1	0.004

**Table 2:** Summary of microstructure cell size of struts.

**Sample**		D1	D2	D3	D4	D5
**Microstructure Cell Size (μm)**	**Range**	0.5–1.0	0.5–1.0	1.0–1.5	1.5–2	1.5–2
	Average	0.75	0.75	1.25	1.75	1.75

**Table 3:** The specific values of porosity level of struts.

**Sample**	D1	D2	D3	D4	D5
**Porosity (%)**	1.87	1.26	0.57	0.12	0.10

**Table 4:** Summary of tensile properties of SLM-fabricated AlSi10Mg struts.

**Sample**	D1	D2	D3	D4	D5
**EM (GPa)**	51.7 (±1.4)	60.5 (±1.2)	65.4 (±1.0)	70.3 (±1.1)	70.4 (±0.8)
**UTS (MPa)**	267.7 (±8.2)	315 (±6.1)	338 (±7.5)	344 (±5.4)	349.5 (±5.9)
**EL (%)**	3.59 (±0.18)	4.26 (±0.2)	5.1 (±0.14)	5.4 (±0.25)	5.37 (±0.14)
**YS (MPa)**	169.6 (±4.5)	203.1 (±3.7)	216.5 (±8)	218.3 (±7.9)	224.3 (±5.9)

## References

[1] Herzog D., Seyda V., Wycisk E., Emmelmann C. (2016). Additive manufacturing of metals. Acta Mater..

[2] Kempen K., Thijs L., Van Humbeeck J., Kruth J.P. (2012). Mechanical properties of AlSi10Mg produced by selective laser melting. Phys. Procedia.

[3] Trevisan F., Calignano F., Lorusso M., Pakkanen J., Aversa A., Ambrosio E.P., Lombardi M., Fino P., Manfredi D. (2017). On the Selective Laser Melting (SLM) of the AlSi10Mg Alloy: Process, Microstructure, and Mechanical Properties. Materials.

[4] Amani Y., Dancette S., Delroisse P., Simar A., Maire E. (2018). Compression behavior of lattice structures produced by selective laser melting: X-ray tomography based experimental and finite element approaches. Acta Mater..

[5] Mazur M., Leary M., Mcmillan M., Sun S., Shidid D., Brandt M. (2017). Mechanical properties of Ti6Al4V and AlSi12Mg lattice structures manufactured by Selective Laser Melting (SLM). Laser Addit. Manuf..

[6] Han X., Zhu H., Nie X., Wang G., Zeng X. (2018). Investigation on selective laser melting AlSi10Mg cellular lattice strut: Molten pool morphology, surface roughness and dimensional accuracy. Materials.

[7] Maskery I., Aboulkhair N.T., Aremu A.O., Tuck C.J., Ashcroft I.A., Wildman R.D., Hague R.J.M. (2016). A mechanical property evaluation of graded density Al-Si10-Mg lattice structures manufactured by selective laser melting. Mater. Sci. Eng. A..

[8] Campoli G., Borleffs M.S., Yavari S.A., Wauthle R., Weinans H., Zadpoor A.A. (2013). Mechanical properties of open-cell metallic biomaterials manufactured using additive manufacturing. Mater. Des..

[9] Campanelli S.L., Contuzzi N., Ludovico A.D., Caiazzo F., Cardaropoli F., Sergi V. (2014). Manufacturing and characterization of Ti6Al4V lattice components manufactured by selective laser melting. Materials.

[10] Delroisse P., Jacques P.J., Maire E., Rigo O., Simar A. (2017). Effect of strut orientation on the microstructure heterogeneities in AlSi10Mg lattices processed by selective laser melting. Scri. Mater..

[11] Hadadzadeh A., Amirkhiz B.S., Odeshi A., Mohammadi M. (2018). Dynamic loading of direct metal laser sintered AlSi10Mg alloy: Strengthening behavior in different building directions. Mater. Des..

[12] Hitzler L., Janousch C., Schanz J., Merkel M., Heine B., Mack F., Hall W., Öchsner A. (2017). Direction and location dependency of selective laser melted AlSi10Mg specimens. J. Mater. Process. Technol..

[13] Thijs L., Kempen K., Kruth J.P., Humbeeck J.V. (2013). Fine-structured aluminium products with controllable texture by selective laser melting of pre-alloyed AlSi10Mg powder. Acta Mater..

[14] Li Z.H., Li B.Q., Bai P.K., Liu B., Wang Y. (2018). Research on the thermal behaviour of a selectively laser melted aluminium alloy: simulation and experiment. Materials.

[15] Wei P., Wei Z.Y., Chen Z., Du J., He Y.Y., Li J.F., Zhou Y.T. (2017). The AlSi10Mg samples produced by selective laser melting: single track, densification, microstructure and mechanical behavior. Appl. Surf. Sci..

[16] Takano N., Takizawa H., Wen P., Odaka K., Matsunaga S., Abe S. (2017). Stochastic prediction of apparent compressive stiffness of selective laser sintered lattice structure with geometrical imperfection and uncertainty in material property. Int. J. Mech. Sci..

[17] Lhuissier P., Formanoir C., Martin G., Dendievel R., Godet S. (2016). Geometrical control of lattice structures produced by EBM through chemical etching: Investigations at the scale of individual struts. Mater. Des..

[18] Suard M., Martina G., Lhuissier P., Dendievela R., Vignat F., Blandin J.J., Villeneuve F. (2015). Mechanical equivalent diameter of single struts for the stiffness prediction of lattice structures produced by Electron Beam Melting. Addit. Manuf..

[19] Nava E.H., Smith C.J., Derguti F., Williams S.T., Léonard F., Withers P.J. (2015). The effect of density and feature size on mechanical properties of isostructural metallic foams produced by additive manufacturing. Acta Mater..

[20] Yan C., Hao L., Hussein A., Raymont D. (2012). Evaluations of cellular lattice structures manufactured using selective laser melting. Int. J. Mach. Tools. Manuf..

[21] Tancogne-Dejean T., Spierings A.B., Mohr D. (2016). Additively-manufactured metallic micro-lattice materials for high specific energy absorption under static and dynamic loading. Acta Mater..

[22] Wang P.D., Lei H.S., Zhu X.L., Chen H.S., Wang C.X., Fang D.N. (2018). Effect of manufacturing defect on mechanical performance of plain weave carbon/epoxy composite based on 3D geometrical reconstruction. Compos. Struct..

[23] Leary M., Mazur M., Elambasseril J., McMillan M., Chirent T., Sun Y.Y., Qiana M., Easton M., Brandt M. (2016). Selective laser melting (SLM) of AlSi12Mg lattice structures. Mater. Des..

[24] Alsalla H., Hao L., Smith C. (2016). Fracture toughness and tensile strength of 316L stainless steel cellular lattice structures manufactured using the selective laser melting technique. Mater. Sci. Eng. A..

[25] Kim T.B., Yue S., Zhang Z.Y., Jones E., Jones J.R., Lee P.D. (2014). Additive manufactured porous titanium structures: Through-process quantification of pore and strut networks. J. Mater. Process. Technol..

[26] Li C.L., Lei H.S., Liu Y.B., Zhang X.Y., Xiong J., Zhou H., Fang D.N. (2018). Crushing behavior of multi-layer metal lattice panel fabricated by selective laser melting. Int. J. Mech. Sci..

[27] Wang Z.Y., Li P.F. (2018). Characterisation and constitutive model of tensile properties of selective laser melted Ti-6Al-4V struts for microlattice structures. Mater. Sci. Eng. A.

[28] Tradowsky U., White J., Ward R.M., Read N., Reimers W., Attallah M.M. (2016). Selective laser melting of AlSi10Mg: Influence of post-processing on the microstructural and tensile properties development. Mater. Des..

[29] Asgari H., Baxtera C., Hosseinkhani K., Mohammadi M. (2017). On microstructure and mechanical properties of additively manufactured AlSi10Mg_200C using recycled powder. Mater. Sci. Eng. A..

[30] Moussaoui K., Rubio W., Mousseigne M., Sultan T., Rezai F. (2018). Effects of selective laser melting additive manufacturing parameters of Inconel 718 on porosity, microstructure and mechanical properties. Mater. Sci. Eng. A..

[31] Hadadzadeh A., Amirkhiz B.S., Li J., Mohammadi M. (2018). Columnar to equiaxed transition during direct metal laser sintering of AlSi10Mg alloy: Effect of building direction. Addit. Manuf..

[32] Qiu C.L., Panwisawas C., Ward M., Basoalto H.C., Brooks J.W., Attallah M.M. (2015). On the role of melt flow into the surface structure and porosity development during selective laser melting. Acta Mater..

[33] Aboulkhair N.T., Everitt N.M., Ashcroft I., Tuck C. (2014). Reducing porosity in AlSi10Mg parts processed by selective laser melting. Addit. Manuf..

[34] Yan C., Hao L., Hussein A., Young P., Huang J., Zhu W. (2015). Microstructure and mechanical properties of aluminium alloy cellular lattice structures manufactured by direct metal laser sintering. Mater. Sci. Eng. A..

[35] Liu L., Kamm P., García-Moreno F., Banhart J., Pasini D. (2017). Elastic and failure response of imperfect three-dimensional metallic lattices: the role of geometric defects induced by selective laser melting. J. Mech. Phys. Solids..

[36] Parthasarathy J., Starly B., Raman S., Christensen A. (2010). Mechanical evaluation of porous titanium (Ti6Al4V) structures with electron beam melting (EBM). J. Mech. Behav. Biomed. Mater..

[37] Qiu C.L., Yue S., Adkins N.J.E., Ward M., Hassanin H., Lee P.D., Withers P.J., Attallah M.M. (2015). Influence of processing conditions on strut structure and compressive properties of cellular lattice structures fabricated by selective laser melting. Mater. Sci. Eng. A..

[38] Weingarten C., Buchbinder D., Pirch N., Meiners W., Wissenbach K., Poprawe R. (2015). Formation and reduction of hydrogen porosity during selective laser melting of AlSi_10_Mg. J. Mater. Process. Technol..

[39] Vrána R., Červinek O., Maňas P., Koutný D., Paloušek D. (2018). Dynamic loading of lattice structure made by selective laser melting-numerical model with substitution of geometrical imperfections. Materials.

[40] Fousová M., Vojtech D., Doubrava K., Daniel M., Lin C.F. (2018). Influence of inherent surface and internal defects on mechanical properties of additively manufactured Ti6Al4V alloy: Comparison between selective laser melting and electron beam melting. Materials.

[41] Öchsner A. (2016). Continuum Damage and Fracture Mechanics.

[42] Aboulkhair N.T., Maskery I., Tuck C., Ashcroft I., Everitt N.M. (2016). The microstructure and mechanical properties of selectively laser melted AlSi10Mg: The effect of a conventional T6-like heat treatment. Mater. Sci. Eng. A..

